# The Level of Anatomical Knowledge, Hard to Establish: a Systematic Narrative Review

**DOI:** 10.1007/s40670-022-01509-w

**Published:** 2022-03-30

**Authors:** Dorothea Maria Koppes, Charlotte Petronella Robertus Triepels, Kim Josephina Bernadette Notten, Carlijn Franscisca Anna Smeets, Rutgerus Franciscus Petrus Maria Kruitwagen, Toon Van Gorp, Fedde Scheele, Sander Martijn Job Van Kuijk

**Affiliations:** 1 Department of Obstetrics and Gynaecology, Maastricht University Medical Centre+, P.O. Box 5800, 6202 AZ Maastricht, The Netherlands; 2 Department of Obstetrics and Gynaecology, Radboud Medical Centre, Nijmegen, The Netherlands; 3 Present Address: GROW-School for Oncology and Developmental Biology, Maastricht University Medical Centre+, Maastricht, The Netherlands; 4 Department of Obstetrics and Gynaecology, Leuven University Medical Centre, Leuven, Belgium; 5 Department of Obstetrics and Gynaecology/Medical Education, OLVG Hospital, Amsterdam, The Netherlands; 6 Department of Medical Education, VU University Medical Centre, Amsterdam, The Netherlands; 7 Athena Institute for Trans-Disciplinary Research, VU University, Amsterdam, The Netherlands; 8 Department of Clinical Epidemiology and Medical Technology Assessment, Maastricht University Medical Centre+, Maastricht, The Netherlands

**Keywords:** Anatomy, Knowledge, Test, Scientific perspectives

## Abstract

**Objective:**

This literature review aimed to gain more insight into the level of anatomical knowledge based on published measurements among medical students, residents, fellows, and specialists.

**Methods:**

We performed an extensive literature search in three online databases: Medline (using PubMed), Web of Science, and Education Resources Information Centre (ERIC).

**Results:**

A total of 30 relevant studies were found. In these studies, participants took different anatomy tests, and their mean/median scaled scores range from 22.5 to 82.4% on a 0 to 100% scale.

**Conclusion:**

This review provides an overview of what is known about measured anatomical knowledge. After critically reviewing the literature, we have to conclude that the existing literature confirms that anatomical knowledge is hard to establish, mainly due to the lack of standardisation.

Further research should focus on ways to define and assess ‘desired anatomical knowledge’ in different contexts. In a next phase, we can discuss if anatomical knowledge is lacking and if interventions are needed.

## Introduction

In 1975, Sinclair wrote an editorial in *The Lancet* expressing his concerns about medical students’ low level of anatomical knowledge [1]. Ever since, many other authors have reported similar concerns [2–8]. In the contemporary literature, clinicians, as well as medical students, report concerns about what they perceive as their own insufficient knowledge of anatomy [9–12]. Some authors even suggest that this lack of anatomical knowledge is the reason why the number of medicolegal claims in healthcare is rising [13, 14]. Anatomical knowledge facilitates learning pathophysiology, supports the examination of a patient, and facilitates rendering a diagnosis [7]. Hence, a good understanding of human anatomy is important not only for surgeons but for all medical specialists to ensure safe clinical practice [7]. Numerous studies describe interventions and education programmes to improve anatomical knowledge, suggesting that there is a need for improvement [15, 16]. However, research on the actual level of anatomical knowledge and the impact of the suggested shortage of anatomical knowledge is scarce. Of the few studies that aim to assess knowledge, many focus on individual opinions instead of on quantification of anatomical knowledge [17].

## Methods

The aim of this review was to gain more insight into the level of anatomical knowledge among medical students, residents, fellows, and specialists by performing a literature review of studies that quantify anatomical knowledge.

The meaning of those findings is discussed from two different scientific perspectives: the deontological one and the utilitarian stance [18].

The deontological perspective is an ethical theory which places special emphasis on the relationship between duty and the morality of human actions. In deontological ethics, an action is considered morally good because of some characteristic of the action itself not because the product of the action is good. The theory believes that ethical actions follow universal moral laws, such as “Don’t lie. Don’t steal. Don’t cheat”. Unlike consequentialism, which judges actions by their results, deontology does not require weighing the costs and benefits of a situation. This avoids subjectivity and uncertainty because you only have to follow set rules. So, following the rules makes deontology easy to apply. But it also means disregarding the possible consequences of our actions when determining what is right and what is wrong.

An example of deontological stance: suppose you are a software engineer and learn that a nuclear missile is about to launch that might start a war. You can hack the network and cancel the launch, but it is against your professional code of ethics to break into any software system without permission. And, it is a form of lying and cheating. Deontology advises not to violate this rule. However, in letting the missile launch, thousands of people will die.

Utilitarianism, a form of consequentialism, is an ethical theory that determines right from wrong by focusing on outcomes. The utilitarian stance holds that the most ethical choice is the one that will produce the greatest good for the greatest number. However, because we cannot predict the future, it is difficult to know with certainty whether the consequences of our actions will be good or bad.

An example of utilitarianism: assume a hospital has four people whose lives depend upon receiving organ transplants: a heart, lungs, a kidney, and a liver. If a healthy person wanders into the hospital, his organs could be harvested to save four lives at the expense of one life. This would arguably produce the greatest good for the greatest number. But, few would consider it an acceptable course of action, let alone the most ethical one.

This study was written in accordance with the Preferred Reporting Items for Systematic Reviews and Meta-Analyses (PRISMA) items that were relevant for this review [19].

### Search

A comprehensive search was performed in the following online databases: Medline (using PubMed), Web of Science, and Education Resources Information Centre (ERIC). We used both medical subject headings (MeSH) and text terms from January 1, 1995, to October 15, 2018. The structured search can be reproduced using the following keywords and logical operators: ((("Students, Medical"[Mesh] OR "Medical students" OR "Medical student" OR "Resident" OR "Residents" OR "Fellow")) AND ("Anatomy/education"[Mesh] OR "Anatomy knowledge" OR "Anatomical knowledge" OR "Clinical anatomy" OR "Anatomy education" OR "Anatomical education")) AND ("Testing" OR "Test" OR "Examination" OR "Test result" OR "Achievement" OR "Cognitive load" OR "Skill" OR "Effectiveness" OR Outcome OR Measurement))).

### Study Selection

Two researchers (D.M.K. and C.S.) selected the studies. First, manuscript titles and abstracts were screened for potential relevance. For all of the selected studies, the full text was reviewed to determine eligibility. In case of disagreement about a study, two other researchers (S.M.J.v.K. and K.N.) decided whether the study was suitable for this literature review or not. We included all studies written in English in which anatomical knowledge was tested among medical students, residents, fellows, or medical doctors.

Over the last decades, anatomy education changed in many universities. Therefore, we chose to exclude any studies conducted before 1995.

In the case of a mixed group of participants (i.e. physician assistants and medical students), only those studies which described the results separately for the different participants were included. From these studies, we only included the participants who fulfilled the inclusion criteria.

**Fig. 1:**
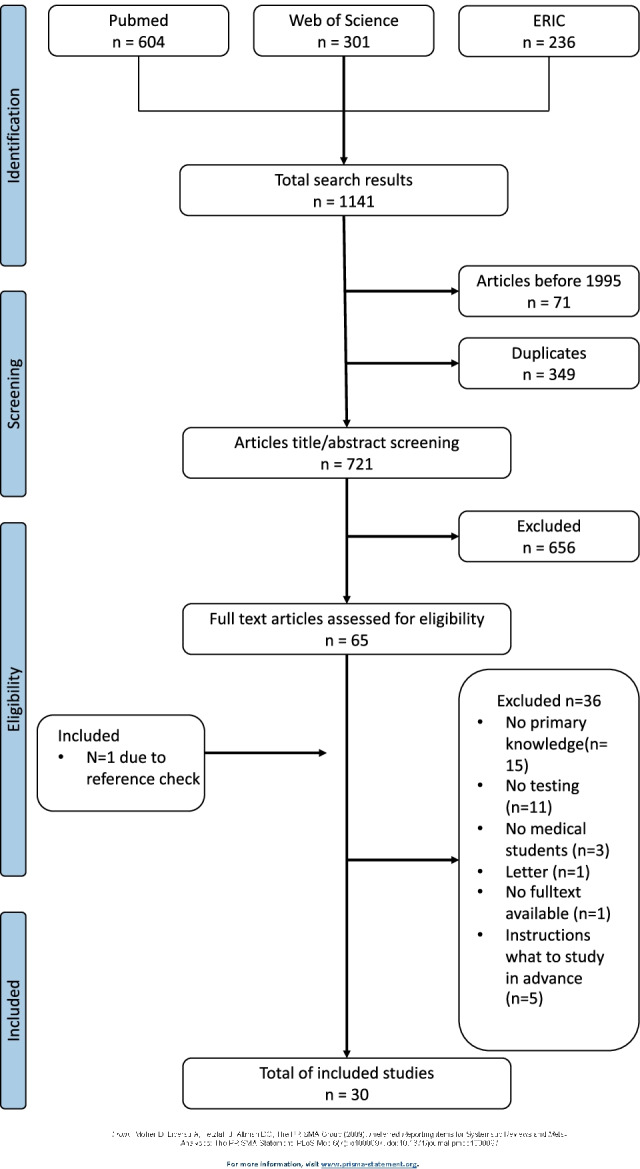
Flowchart of literature search

The flowchart of the literature search is shown in Fig. 1.

### Scaled Score

We anticipated heterogeneity with respect to the quantification of the test results between the different studies and within the studies by using different scales or scores. In order to aid interpretation, all the scales were recalculated to a scaled average test score with a range instead of the SD between 0 and 100%.

## Results

### Study Selection

The flowchart of the literature search is shown in Fig. 1.

The electronic search strategy identified 1141 studies, which were assessed for eligibility. After the exclusion of duplicates and studies conducted before 1995, 721 studies remained eligible. Titles and abstracts were screened for eligibility, and 65 articles were selected for further reading. After full-text reading, 29 articles were selected for inclusion. A cross-reference search of the references of the included articles resulted in one additional relevant article. A total of 30 articles were included.

### Study Characteristics

Details of the included studies are summarised in Tables 1 and 2.

**Table 1:** Anatomical knowledge [20, 21, 26, 27, 30, 33, 34, 38–41]

**Author (year)**	**Participants (*n*)**	**Study design**	**Anatomical region**	**Measurement method**	**Result of study**	Scaled score 0–100*	**Remarks of the authors**
Brunk et al. (2017) [20]	5th- and 6th-year medical students (5383)	Cohort (multicentre)	All anatomy	Anatomical multiple-choice questions (Berlin progress test)	Factual knowledge score 40.8% Simple application 38.3% Clinical application 22.5%	Factual knowledge 40.8% (no SD known) Simple application 38.3% (no SD known) Clinical application 22.5% (no SD known)	Five panels of experts set a standard score for fail/pass for each of the three domains. Those scores were, respectively, 67.6%, 73.5%, and 53.5%
Holda et al. (2018) [30]	Medical students (931) and medical graduates (interns, residents, specialists) (255)	Cross-sectional	All anatomy	Internet-based survey with 10 open and 10 multiple-choice questions of labelled structures on cadaveric specimens	Mean score 65.6% Mean score students 67.3% Mean score graduates 59.5%	Mean score 65.6% Mean score students 67.3% Mean score graduates 59.5%	The cutoff for fail/pass was set at 60%. A total of 27.9% did not pass the test. The overall mean score is moderate according to the authors
Prince et al. (2005) [21]	Fourth-year medical students (348)	Cohort (multicentre)	Clinical anatomy	107 questions which were linked to 13 patient cases Test consisted of open questions, multiple-choice questions, and true/false questions	Mean score 53.2% (range 32–80)	Mean score 53.2% (range 32–80)	Four different panels, consisting of fourth-year students, recent graduates, clinicians, and anatomists established what in their opinion was a standard score. Those standard scores were, respectively, 56.0%, 46.9%, 54.3%, and 50.2%
Jurjus et al. (2014) [27]	Third-year medical students (189)	Cohort	General surgery and obstetrics and gynaecology	20-question test 1 week prior to obstetrics/gynaecology clerkship 25-question test 1 week prior to general surgery clerkship. 75% multiple-choice questions and 25% image labelling questions	Surgery rotations 67.0% (range 62.1–70.6) Ob-gyn rotations 64.4% (range 63–69.7)	Surgery rotations overall 67.0% (range 62.1–70.6) Ob-gyn rotations overall 64.4% (range 63–69.7)	
Doomernik et al. (2017) [38]	Second-year medical students (165)	Cross-sectional	Abdominal anatomy	53 items correlated to clinical cases and computed tomography images	Mean score 37.9 (SD 5.48) Relative score 71.5% (10.3%)	71.5% (range 61.2–81.8)	
Grunfeld et al. (2012) [39]	Graduating medical students (134)	Cohort (multicentre)	Musculoskeletal	75 questions, consisting of 14 basic science and 61 clinical science Questions were selected from the National Board of Medical Examiners Musculoskeletal Subject Examination	Medical students 73.8% (SD 9.7)	73.8% (range 64.1–83.5)	
Diaz-Mancha et al. (2016) [26]	Medical students (39)	Cross-sectional	Carpal and tarsal bones	Recognising labelled bone structures, 15 in total	Medical 6.1/15 (SD 3.27)	40.9% (range 19.1–62.7)	
Dickson et al. (2009) [34]	Accident and emergency senior house officers (26)	Cohort (multicentre)	Hand anatomy	11 questions; one question about hand bones, 5 questions about tendons, and 5 about nerves	Overall score 26.9%	Overall score on all questions 26.9%	
Gupta et al. (2008) [40]	Preregistration house officers (29) Senior house officers (68) Specialist registrars (21)	Cohort	All anatomy	Multiple-choice questions covering 15 areas of the body	PHO 72.1% (SD 3.29) SHO 77.1% (SD 2.16) Specialist registrars 82.4% (SD 2.17)	PHO 72.1% (range 68.8–75.4) SHO 77.1% (range 74.9–79.3) Specialist registrars 82.4% (range 80.2–84.6)	
Navarro-Zarza et al. (2014) [41]	Rheumatology fellows (84) Rheumatologist (61) Non-rheumatologists (25)	Cohort (multicentre)	Joints	20 questions selected from a pool of 40 anatomic items	Rheumatology fellows 50.8 (SD 17.6) Rheumatologists 44.3 (SD 17.9) Non-rheumatologists 39.1 (SD 17.6)	Rheumatology fellows 53.5% (range 34.9–72.0) Rheumatologists 46.6% (range 27.8–65.5) Non-rheumatologists 41.2% (range 22.6–59.7)	
Mizrahi et al. (2017) [33]	Gynaecology residents (52)	Cross-sectional	Pelvic anatomy	Questions and image-labelling questions, 20 questions in total	Overall score 6.67 (SD 0.46) Global score youngest (yr 1–3) 5.53 (SD 0.46) Global score eldest (yr 4–5) 9.24 (SD 0.76)	Overall 33.4% (range 31.1–35.7) Youngest 27.7% (range 25.4–30.0) Eldest 46.2% (range 42.4–50)	Ob-gyn resident’s level in anatomy is poor, and residents should be educated to specific teaching in anatomy throughout their residency program

The primary aim of the included studies was to quantify current anatomical knowledge

**Table 2:** Intervention studies [23–25, 28, 29, 31, 32, 42–53]

**Author (year)**	**Participants (*n*)**	**Study design**	**Anatomical region**	**Measurement method**	**Result of study**	Scaled score 0–100*	**Remarks of the authors**
Jurjus et al. (2016) [42]	Third-year medical students during clerkship ob-gyn (143)	Cohort	Gynaecology	Test consisting of *22 multiple-choice questions in e-learnings *25 multiple-choice questions and matching questions in a laboratory session	All questions pretest mean 59.5% (SD 2.09)	All questions 59.5% (range 57.4–61.6)	
Maresky et al. (2018) [﻿^52^]	First-year medical students (59)	Cohort	Cardiac anatomy	5 conventional cardiac anatomy questions 5 visual-spatial questions	Overall score 50.9% (SD 16.5) Conventional cardiac anatomy 62.9% Visual-spatial cardiac anatomy 38.6%	Overall score 50.9% (range 34.4–67.4) Conventional cardiac anatomy 62.9% Visual-spatial cardiac anatomy 38.6%	
Luetmer et al. (2017) [53]	First-year medical students (53)	Cohort	Shoulder and elbow	Six clinical scenarios in the form of multiple-choice questions	Median score 67%, mean 66.1 (SD 13.9)	Median score 67% (range 53.1–80.9)	
Morgan et al. (2017) [43]	Fourth-year medical students Applied clinical anatomy (47) Surgery resident preparation course (40) Obstetrics and gynaecology course (36)	Cohort	Musculoskeletal system, emergency medical procedures, and radiology	Three applied clinical anatomy courses with a pretest on physical examination, anatomical knowledge, and radiology	Emergency medical procedure 45.9% (SD 12.77) Musculoskeletal system 56.9% (SD 14.6) Obs and gyn 67.3% (SD 18.19)	Emergency medical procedure 45.9% (range 33.18–58.72) Musculoskeletal system 56.9% (range 42.33–71.53) Obs and gyn 67.3% (range 49.14–85.52)	All of the intervention courses emphasised the correlations between anatomical concepts and clinical applications. Thus, the applied clinical anatomy course was divided into three separate courses: emergency medical procedures, anatomy meets radiology, and the musculoskeletal system. The knowledge of the participants was assessed through a test compromised of questions created by the American Association of Anatomists and a question bank created by one of the course directors
Burgess et al. (2012) [24]	Stage-3 senior medical students (42)	Cohort	All anatomy	Identify 20 labelled structures in four wet specimens of different anatomical regions	Pretest median is 9/20 (range 2–18)	45% (range 10–90)	
Sarkis et al. (2014) [44]	Final-year graduate medical students (24)	Cohort	All anatomy	Identify 20 labelled structures located over four wet specimens	Premedian 8/20 (range 2–14)	40% (range 10–70)	
Stott et al. (2016) [45]	Medical students’ years 3–5 (18)	Cohort	Heart	20 multiple-choice questions, consisting of a mixture of basics and clinical science	Pre-course score 59.6% (SD 13.8)	59.6% (range 45.8–73.4)	
Mackenzie et al. (2017) [46]	Surgical residents’ year 3–6 (40)	Cohort (multicentre)	Emergency medicine	Assessment done by 1 anatomist and 1 physician located in the same laboratory with a standardised script	Pretest anatomy score 47% (SD 11)	47% (range 36–58)	
Jaswal et al. (2015) [47]	Radiation oncology residents (29)	Cohort (multicentre)	All anatomy and radiology	30 item multiple-choice question style test. Each question consisted of 1 or more images projected on a large screen along with the question. Each question was restricted to 15 s, with no opportunity to revisit previous questions	Pretest median 18.2/30 (range 16–21)	60.6% (range 53–70)	
Burgess et al. (2016) [23]	Postgraduate surgical trainees (26)	Cohort	All anatomy	Standardised practical examination of 20 items	Pretest median 8/20 (range 5–14)	40% (range 20–75)	
Ozcan et al. (2015) [48]	Urology residents (25)	Cohort (multicentre)	Kidney, ureter, retroperitoneal region, prostate, bladder, urethra, pelvis, penis, and scrotum	20 multiple-choice questions with a maximum of five alternative answers. Questions were randomly selected from a bank of multiple-choice questions prepared by 37 scientists	Pretest median 11.7/20	58.5%	
Corton et al. (2003) [25]	Residents (24)	Cohort	Pelvis	The practical exam consisted of identifying 20 tagged structures on prosected specimens The written exam consisted of 20 multiple-choice questions that assessed residents’ knowledge of perineal, retropubic, presacral, retroperitoneal, pelvic support anatomy, and clinical correlations	Practical exam Overall correct score 72% Written exam Overall correct score 58.5%	Practical exam Overall correct score 72% Written exam Overall correct score 58.5%	No standard deviation or range was given
Arantes et al. (2017) [32]	General practitioner trainees (20)	Cohort	Neuroanatomy	30 identification questions 30 multiple-choice questions referring to clinical cases	Overall mean identification score 26.8% Overall mean multiple-choice score 56.7%	Overall mean identification score 26.8% Overall mean multiple-choice score 56.7%	
Juo et al. (2018) [31]	Surgical interns (14)	Cohort	All anatomy	30 multiple-choice questions 20 structure identification questions	Average multiple-choice score 15.9 (SD 5.1) 53% Average identifications score 10.1 (SD 3.0) 50.5%	Average multiple-choice score 53% (range 36–70%) Average identifications score 50.5% (range 35.5–65.5%)	
Chino et al. (2011) [49]	Postgraduate radiology oncology residents years 2–5 (10)	Cohort (multicentre)	All anatomy and radiation oncology	10–15 question test consisting of boards-style multiple-choice questions, segmentation of radiographic images of critical tissues, and radiation field design	Median pre-test score 66% (range 53–82) board test MCQ pretest median 71% (range 41–100)	Median pretest score 66% (range 53–82) board test MCQ pretest median 71% (range 41–100)	
Saavedra et al. (2016) [28]	Rheumatology fellows (17) Orthopaedic fellows (14)	Cohort (multicentre)	Joints and musculoskeletal	20 questions of identification or demonstration of relevant anatomical items (or their action), arranged by regions and asked in five dynamic stations	Median correct answers pretest Orthopaedic 7/20 (range 2–12) Rheumatology 5/20 (range 1–10)	Orthopaedic 35% (range 10–60) Rheumatology 25% (range 5–50)	
Barton et al. (2009) [50]	10 gynaecological oncologist fellows	Cohort (multicentre)	Vulva, vagina, perineum, anterior and posterior abdominal wall, retroperitoneum, groin, pelvis, abdomen, and, radiological anatomy	Multiple-choice questionnaire on abdominal and pelvic anatomy	Mean 57/100 (range 32–71)	57% (range 32–71)	
Corton et al. (2006) [51]	Medical students and postgraduate year 1–4 (36) Female pelvic medicine and reconstructive surgery fellows (3)	Randomised longitudinal cohort	Pelvis	20 questions about anatomy pelvic support and 36 multiple-choice questions about the vulva and perineal anatomy	Pretest pelvic support -Interactive 56.0 (SD16.9) -Conventional 53.4 (SD 13.4) Vulvar and perineal -Interactive 63.2 (SD 9.1) -Conventional 61.8 (SD 17.7)	Pretest pelvic support -Interactive 56.0% (range 39.1–72.9) -Conventional 53.4% (range 40–66.8) Vulvar and perineal -Interactive 63.2% (range 54.1–72.3%) -Conventional 61.8% (44.1–79.5%)	
Labranche et al. (2015) [29]	Medical physicists (3) Fellow (1) Radiation oncology residents (13)	Cohort	Thorax, abdomen, male pelvis, and female pelvis	10 multiple-choice questions and identification questions	Thorax 4.5/10 (SD 2.6) Abdomen 5.1/10 (SD 2.1 Male pelvis 6.1/10 (SD 1.4)	Thorax 45% (range 19–71) Abdomen 51% (range 30–72) Male pelvis 61% (range 47–75)	

Included studies evaluate intervention and tested anatomical knowledge before and after the intervention. For this review, we assumed that the pre-intervention tests reflected the participants’ level of anatomical knowledge. So, only the pre-intervention score is included

Table 1 shows studies whose primary aim was quantifying current anatomical knowledge. Eleven studies primarily evaluated anatomical knowledge. There were six studies which primarily evaluated the anatomical knowledge of medical students, and four which evaluated (young) medical doctors. One study assessed the anatomical knowledge of fellows and medical specialists.

The nineteen studies summarised in Table 2 evaluated an intervention and tested anatomical knowledge before and after the intervention. For this review, we assumed that the pre-intervention tests reflected the participants’ level of anatomical knowledge. Hence, we only extracted the pre-intervention results from these studies. Seven studies tested the knowledge of medical students before the intervention by the authors. A total of eight intervention studies involved a pretest on residents. We identified two studies which performed an intervention study on fellows. Two intervention studies focused on the anatomical knowledge of a mixed group of students, residents, and fellows. Table 3 shows test results based on different types of questions, subdivided into multiple choice, board style, and fill in the blank.

**Table 3:**
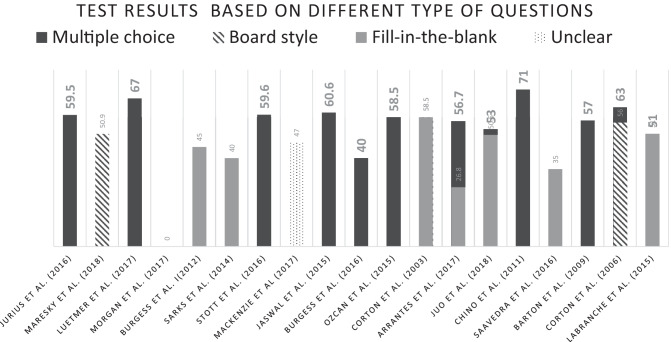
Test results based on different types of questions. Subdivided into multiple choice, board style, and fill in the blank. Scaled score 0–100%

## Discussion

### Main Findings

The actual measured knowledge of anatomy of medical students, residents, fellows, and specialists differed substantially between studies. Scores were reported as median or mean and after scaling ranged from 22.5 to 82.4% correct answers. Scores per group were 22.5–73.8% for medical students, 26.9–82.4% for residents, and 25.0–63.2% for medical doctors/fellows. Almost two-thirds of the total given mean/median scores were below 60%.

In six of the thirty studies, the authors expressed their interpretation of the measured level of anatomical knowledge. Based on the measurement results of their research, they conclude that the level of knowledge is deficient and moderate to worryingly low.

### Interpretation of the Findings

The question of whether knowledge of anatomy is sufficient or too low may be approached from different perspectives. One of those perspectives is the deontological one. As a physician, one has to have good knowledge of anatomy. It is an obligation or duty towards the patient and is a generally accepted rule we should conform to. The current literature seems to lean on deontological ethics. The opinion provided by five of the studies that knowledge is moderate to worrying low is an example of deontology. However, there is no research on what the level of knowledge should be. The utilitarian stance is another perspective we can approach. Utilitarianism states that the best action is the one that maximises utility, which is usually defined as that which produces the greatest well-being of the greatest number of people.

When is anatomical knowledge worryingly low so that it will cause danger to a patient? Or the opposite, that it leads to higher appreciation of the patient? We could not find any evidence showing that a low level of anatomical knowledge is the reason for medical errors or unsatisfied patients.

This might suggest that the way most medical professionals deal with anatomy is pragmatic and a fair choice in the abundance of demanded competencies. However, we must also acknowledge that the absence of proof is not always the proof of absence.

### The Quest for a Gold Standard for How Much Anatomy

So far, the literature does not provide a convincing gold standard for how much anatomy is required for safe clinical practice. Following the deontological stance, an international consensus could set the standard. However, with more than 100 curricula all over the world, this sounds like an impossible job. Two studies, Brunk et al. and Prince et al. tried to set a gold standard through the use of expert panels. In the study of Brunk et al. the pass rate was set at 60.4% for 5th- and 6th-year medical students. The actual score was 29.9%. Prince et al. used different expert panels to set the standard, showing that fourth-year students set the highest pass rate at 56.0%, whereas recent graduates set the lowest pass rate at 46.9%. The mean overall score was 53.2%. The conclusion of the authors of both studies was that the results are way below the expected standard [20, 21]. However, given the known retention levels of basic science, it is questionable whether this conclusion is correct. In an extensive study by Custers et al. it is shown that participants still in medical school and those not too long out of it achieved scores of approximately 40% correct answers on basic science. Specifically looking at anatomical knowledge for 5th- and 6th-year students, this percentage lies between 45 and 50% [22].

### Strengths and Limitations

Our review holds some limitations that need to be addressed. We included 30 studies in which 30 different tests were used. There was much heterogeneity in the number and type of questions, as well as in the region of interest which was tested. Based on the different characteristics of the tests, some can be regarded as more reliable than others. One of the most frequent manners of testing was the identification of labelled structures with a maximum of 20 items [23–33]. But Brunk et al. used the Berlin Progress Test Medicine (PTM), a test of 200 items chosen from an item pool containing 5000 items. All items are administered in single-best answer multiple-choice format and typically make use of clinical vignettes [20]. In contrast, Dickson et al. derived their conclusion on an 11-question test [34]. Besides, the sort of test, the context in which it was taken, the time between the test and the period in which the anatomy was learned, and if there has been repeated learning are important variables. In our selection, we only included studies that did not test anatomical knowledge after an intervention or repeated learning. The time interval between the moment the material was studied and when it was tested was hard to assess since there are many different curricula. However, in most curricula, anatomy is taught in the preclinical years.

This diversity of tests and moment of testing creates two difficulties. First, although pooling the results using meta-analysis techniques is statistically not impossible, we felt it would not yield a useful summary of test results for the purpose of our study. Second, it makes it hard to interpret the reliability of the scores. For example, an average score of 50% on a difficult exam with questions of function and applied clinical anatomy might be the same as a 90% score on an easy exam with only identification of structures.

Another point to mention is the diversity of participants. In the included study, this ranged from medical students up to medical doctors. This can be seen as a limitation if comparisons between studies are made, but it is also a strength in providing some insight into anatomical knowledge over time and making the results of our review generalizable to a broader group.

The strength of our study can be found from a more philosophical point of view. Our review has shown that anatomical knowledge is hard to establish, and a gold standard cannot be found. The questions around anatomy education should be rephrased using different paradigms from philosophy. The main question will be “when to give students the right level and amount of which anatomy in order to feel safe and competent to do their clinical work”. This means that we should also focus on ways to define and assess this level.

### Suggestions and Challenges for the Future

In our search for the level of anatomical knowledge, the result is the absence of standardisation. Not only in ways of testing but also in need to know knowledge. Without agreements about the need for knowledge, which will differ at different stages of medical and postgraduate education, it is difficult to judge the level of anatomical knowledge. There are universities with an extensive curricular plan including a good description of what anatomy knowledge is expected [35]. This is a good start, although it can vary from university to university and from country to country. While in general, the human being and her anatomy and illness do not vary. A suggestion to remedy this absence is to conduct a Delphi study to determine what knowledge is required to know. In a Delphi study, experts will discuss a topic and reach a consensus. An example is being carried out in the Netherlands for the gynaecology speciality [36]. After focus groups, in-depth interviews, and two Delphi rounds, a core list of anatomical structures that are relevant to the safe and competent practice of general gynaecologists was identified. Such a list can be used to guide gynaecology postgraduate education and assessment.

The second challenge is the wide variety of specialities and subspecialities. A gastrointestinal surgeon does not need to have the same knowledge as a cardiac surgeon or a gynaecologist. Determining what the knowledge need for each stage of education, speciality, and subspecialty and what the need to know knowledge is will be an extensive job. However, in our opinion, this is an indispensable step in the process of assessing and determining anatomical knowledge.

A third challenge is the way of testing. Our results already show different ways in which anatomical knowledge can be assessed. In general, anatomical knowledge can be tested using a variety of assessment tools, such as multiple-choice exams, oral exams, or structured practical examinations. These tools reflect the three domains of anatomy training: theoretical knowledge, practical 3D application of this knowledge, and clinical or bedside application of knowledge [37]. So, after determining which knowledge is essential, this anatomical knowledge should be tested in various ways within the different domains.

## Conclusion

This review provides an overview of what is known about measured anatomical knowledge. After critically reviewing the literature, we have to conclude that the existing literature confirms that anatomical knowledge is hard to establish, mainly due to the lack of standardisation.

Further research should focus on ways to define and assess “desired anatomical knowledge” in different contexts. Suggestions are to conduct a Delphi study among experts from the field to define essential anatomical structures. After that, it is important to assess anatomy knowledge through various assessments to test different domains of anatomical knowledge. In a next phase, we can discuss if anatomical knowledge is lacking. And if so, what the impact of this shortage is and whether interventions are needed.

## Data Availability

All data generated or analysed during this study are included in this published article (and its supplementary information files).

## Abbreviations

S.M.J.v.K.: Sander Martijn Job van Kuijk
K.N.: Kim Josephina Bernadette Notten
ERIC: Education Resources Information Centre
